# Deletions in Genes Participating in Innate Immune Response Modify the Clinical Course of Andes Orthohantavirus Infection

**DOI:** 10.3390/v11080680

**Published:** 2019-07-25

**Authors:** Grazielle Esteves Ribeiro, Luis Edgardo Leon, Ruth Perez, Analia Cuiza, Pablo Agustin Vial, Marcela Ferres, Gregory J. Mertz, Cecilia Vial

**Affiliations:** 1 Programa Hantavirus, Instituto de Ciencias e Innovación en Medicina, Facultad de Medicina, Clínica Alemana Universidad del Desarrollo, Av. La Plaza 680, Las Condes, Región Metropolitana 7500000, Chile; 2 Instituto de Ciencias Biomédicas, Facultad de Ciencias de la Salud, Universidad Autónoma de Chile, Pedro de Valdivia 425, Región Metropolitana 7500000, Santiago, Chile; 3 Departamento Pediatria Clinica Alemana de Santiago, Av Vitacura 5951, Vitacura, Región Metropolitana 7500000, Chile; 4 Departamento de Enfermedades Infecciosas e Inmunología Pediátricas, Pontificia Universidad Católica de Chile, Marcoleta 391, Santiago, Región Metropolitana 7500000, Chile; 5 Department of Internal Medicine, MSC10 5550, 1 University of New Mexico, Albuquerque, NM 87131-0001, USA

**Keywords:** HCPS, ANDV, hantavirus

## Abstract

Andes orthohantavirus (ANDV) is an important human pathogen causing hantavirus cardiopulmonary syndrome (HCPS) with a fatality rate of 30% in Chile. Around 60% of all cases have a severe clinical course, while the others have a mild clinical course. The main goal of this study was to understand if the genetic variation of patients is associated with the clinical course they develop after ANDV infection. For this, the frequency of copy number variants (CNVs, i.e., deletions and duplications) was studied in 195 patients, 88 with mild and 107 with severe HCPS. CNVs were called from intensity data of the Affymetrix Genome-Wide SNP Array 6.0. The analysis of the data was performed with PennCNV, ParseCNV and R softwares; Results: a deletion of 19, 416 bp in the q31.3 region of chromosome 1 is found more frequently in severe patients (*p* < 0.05). This region contains Complement Factor H Related (*CFHR1)* and *CFHR3* genes, regulators of the complement cascade. A second deletion of 1.81 kb located in the p13 region of chr20 was significantly more frequent in mild patients (*p* < 0.05). This region contains the *SIRPB1* gene, which participates in the innate immune response, more specifically in neutrophil trans-epithelial migration. Both deletions are associated with the clinical course of HCPS, the first being a risk factor and the second being protective. The participation of genes contained in both deletions in ANDV infection pathophysiology deserves further investigation.

## 1. Introduction

Hantavirus cardiopulmonary syndrome (HCPS) is a hemorrhagic fever caused by “New World” hantaviruses, which are endemic to North and South America [1]. HCPS is caused by different hantavirus species that are characteristic for each region: Sin Nombre orthohantavirus (SNV) is the most common cause of HCPS in the United States, while Andes orthohantavirus (ANDV) is the etiologic agent of HCPS in Chile and in southern Argentina. Both viruses are similar in regard to genome identity and clinical presentation. Transmission to humans occurs following human exposure to infected rodents of the Muridae family [2]. ANDV is the only hantavirus that is also capable of transmission from person to person [3,4].

Although the same virus infects people in Chile and southern Argentina, the clinical course varies by individual. After the incubation period that ranges from 10 to 40 days [5] and a febrile prodromal stage, a cardiopulmonary phase starts with the development of a dry cough. A severe clinical course may develop within hours with respiratory distress requiring mechanical ventilation, bilateral pulmonary infiltrates, severe shock requiring vasoactive drugs and death in one-third of the cases [6,7]. Not all the people who acquire ANDV infection develop this severe disease [8]. Some cases do not reach the cardiopulmonary phase or develop only mild respiratory distress. Seroprevalence rates of ANDV in Chile range between a country-wide average of 0.27% up to 2.5% in a rural community in region IX, suggesting that asymptomatic infection, although infrequent, also occurs [9,10]. The reason for this difference in clinical course is unknown.

The pathophysiology of HCPS is not fully understood, but primary features of hantavirus infection include thrombocytopenia, microvascular leakage causing pulmonary edema, and cardiogenic shock [11]. Hantaviruses have no direct cytopathic effect, which suggests that host-related immune mechanisms such as an exacerbated immune response and a cytokine storm induced by the infection might be involved in the pathogenesis [12,13,14]. Moreover, a recent study analyzing the inflammatory response of these patients showed that inflammation associated serum markers were significantly increased in HCPS patients, including IL-1β, IL-6, IL-10, IL-18, TNF, IFN-γ, BAFF, complement proteins C5/C5a, and granzyme A and B [15].

Several studies have suggested that host genetic constitution may influence the clinical outcome of hantavirus infection, resulting in mild or severe disease [16,17,18,19,20,21]. In HCPS patients, HLA-B*08 and HLA-DRB1*15 alleles were associated with disease severity, as well as polymorphisms in the *IL28B* gene [17,22].

Copy number variants (CNVs) are one of the major sources of human genetic variation [23]. CNVs are defined as a DNA segment of one kilobase (kb) or larger, present at variable copy number across the genome of different individuals (i.e., deletions or duplications) [24,25]. They can potentially affect a phenotype due to a gain or loss of gene function. Diverse CNVs have been associated with susceptibility of infectious diseases such as malaria or HIV [24,26,27]. Nevertheless, no association study has been done for CNVs and hantavirus infection.

The goal of this study was to identify, in an unbiased manner, CNVs associated with disease severity in patients infected with ANDV. The results showed two deletions associated with the clinical course of HCPS. The first deletion, which was more frequent in severe patients, contains *CFHR1* and *CFHR3* genes coding for complement factor H related proteins (CFHRs) 1 and 3, which can regulate the complement pathway. The second deletion was found more frequently in mild patients and contains signal-regulatory protein beta 1 (*SIRPB1*) gene, which participates in innate immune response and induces neutrophil epithelial transmigration.

## 2. Materials and Methods

### 2.1. Sample Collection and Genotyping

For this study, two types of patients were considered: (1) Patients enrolled in previous studies who had samples stored (*n* = 81). (2) Patients enrolled prospectively specifically for this study (*n* = 114). In both groups there was a follow-up of the clinical course and outcome. Study participants were classified according to the following clinical outcomes: Severe: Patients who required vasoactive drugs and mechanical ventilation (this group includes all patients that died); Mild: Patients who had only prodromal symptoms or had mild cardiorespiratory involvement without the need for mechanical ventilation or vasoactive drugs. Overall 195 ANDV infected patients were included (107 severe and 88 mild), and ANDV infection was confirmed by serology or RT-qPCR. Patients were enrolled in 1 research centers from the central and southern regions of Chile between 2006 and 2014. Local Ethics Committees at each participating institution approved the study (Clínica Alemana Universidad del Desarrollo IRB4858, FWA8639, 22 June 2011; Hospital Clínico UC, IRB2886, FWA4080, 22 June 2011; Clínica Santa María IRB8649, FWA18731, 22 August 2012; Clínica Las Condes, IRB8758, FWA18748, 5 October 2013; Hospital Chillán, IRB1914, FWA 3412, 5 April 2014; Hospital Regional de Temuco, IRB 6293, FWA 3412, 10 April 2012; Hospital Base Valdivia IRB1914 FWA 3412, 28 February 2013, Hospital Base Osorno IRB1914 FWA 3412, 7 April 2012; Hospital Regional Puerto Montt, IRB1914, FWA 3412, 26 November 2012, Hospital Regional Coyhaique, IRB1914, FWA 3412, 3 April 2013), and informed consent was obtained from all participants, their parents, or legal guardians. Stored samples were anonymized and used with the approval of the institutional Ethics committee at Facultad de Medicina, Clinica Alemana, Universidad del Desarrollo (IRB4858, FWA8639, 8 March 2012).

### 2.2. Genotyping with Affymetrix SNP 6.0 Array

Genomic DNA was extracted from whole blood by using AxyPrep blood genomic DNA kit (Axygen, Corning, NY, USA). Genotyping was performed by using Affymetrix Human SNP 6.0 array (Affymetrix, Santa Clara, CA, USA) according to the manufacturer’s recommendations. Affymetrix Human SNP 6.0 array contains 1.8 million genetic markers and more than 946,000 non-polymorphic CNV probes. Affymetrix array scanner 3000 7G (Thermo Fisher Scientific, Waltham, MA, USA) was used to quantify the fluorescence intensity and Affymetrix Gene Chip Console (AGCC) software (Thermo Fisher Scientific) was used for initial quality controls checks.

### 2.3. CNV Calling and Filters

CNV calling and filters was done as described [28,29,30]. Briefly, samples that passed standard SNP quality control (QC) procedures according to AGCC software were used in the CNV analysis pipeline. These samples (*n* = 195) were divided into two groups according to disease severity: severe (*n* = 107) and mild (*n* = 88). The Log R ratio (LRR) and B allele frequency (BAF) measures were calculated with the Affymetrix Power Tools software version 1.16.0 (https://www.affymetrix.com/support/developer/powertools/changelog/index.html). CNV calling was performed with the PennCNV software standard procedure, using the standard hg18 “all” PennCNV hidden Markov model (HMM), GC model correction and population frequency of B allele (pfb) files for the Affymetrix SNP 6.0 arrays [28].

Stringent quality control was applied to all samples, with a Log R Ratio (LRR) standard deviation of 0.35 as cutoff, as suggested [31]. Also, samples that failed QC after the wave adjustment were removed. Additionally, samples containing more than 100 CNV calls were also excluded, because PennCNV frequently generates a high number of CNV calls for samples with very low quality even if LRR standard deviation appears normal. A total number of 111 samples (61 severe and 50 mild) passed the filters. In order to avoid potential CNV-calling errors, centromeric and telomeric regions were removed from the analysis by delimiting them to a 500 kb window. Also, genomic regions encoding immunoglobulin genes, which have been previously shown be potential sites of false-positive CNV calls, were removed using their corresponding genomics coordinates as suggested by the PennCNV software website [28]. Finally, the UCSC Genome Browser LiftOver tool was used to convert CNV coordinates to the GRCh38 build [32].

### 2.4. Statistical Analysis

IBM SPSS Statistics v.20 was used for the analysis of patients’ demographic characteristics. A Shapiro normality test was performed and a t-test was used for studying association between age and clinical course; Fisher exact test was used for gender analysis. Number and size of CNVs (deletions and duplications) in severe and mild samples were counted for CNV burden analysis. For this, a normality test was done using the Shapiro–Wilk test, and a Wilcoxon rank sum test was employed to compare CNV burden data between groups (R software v3.2). The association analysis comparing CNV frequencies between severe and mild patients was done with ParseCNV [33], using 1000 permutations for main parameters. The Odds ratio (OR) and confidence interval (CI) of the CNVs were calculated using the epitools package in Rv3.2. A *p*-value < 0.05 was considered significant. OR > 1 considered CNV to be associated with higher odds of having a severe clinical course.

## 3. Results

### 3.1. ANDV Infected Patient Socio-Demographic Characterization

Table 1 shows the patients’ demographic characteristics and that they do not have a statistical difference between the two groups (*p*-value > 0.05).

### 3.2. CNV Analysis

**Figure 1:**
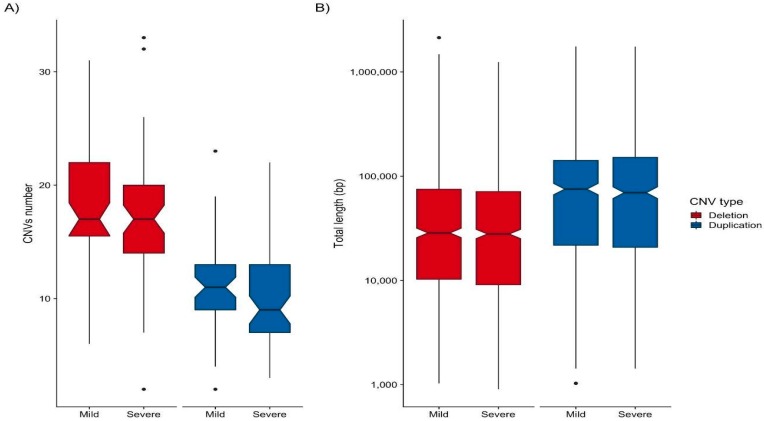
Distribution of total copy number variant (CNV) burden in hantavirus infected patients. A standard box and whiskers plot showing distribution of number and length of CNV for deletions (red) and duplications (blue) in mild and severe patients. Colored boxes are the first and third quartiles for each distribution, median value is represented by horizontal lines and dot points are outliers.

To study the potential association of patients’ CNVs with HCPS disease severity, we analyzed 195 ANDV infected patients, who were classified according to their clinical course: severe (*n* = 107) and mild (*n* = 88). After quality control (QC) of genotyping data, 111 samples were retained in the analysis (60 with a severe clinical course). CNVs were called for each of these samples. In order to assure that CNVs’ characteristics (amount, size and type) are not a confounding variable in severe and mild cases, a global CNV burden test was performed. The number of CNVs (median 13 for severe versus 14 for mild) and length of CNVs (median 34.440 kb for severe versus 36.917 kb for mild) in both groups was not statistically significant. The same analysis was done splitting data into CNV type: deletions and duplications, and no statistical difference was found between severe and mild group (Figure 1).

**Figure 2:**
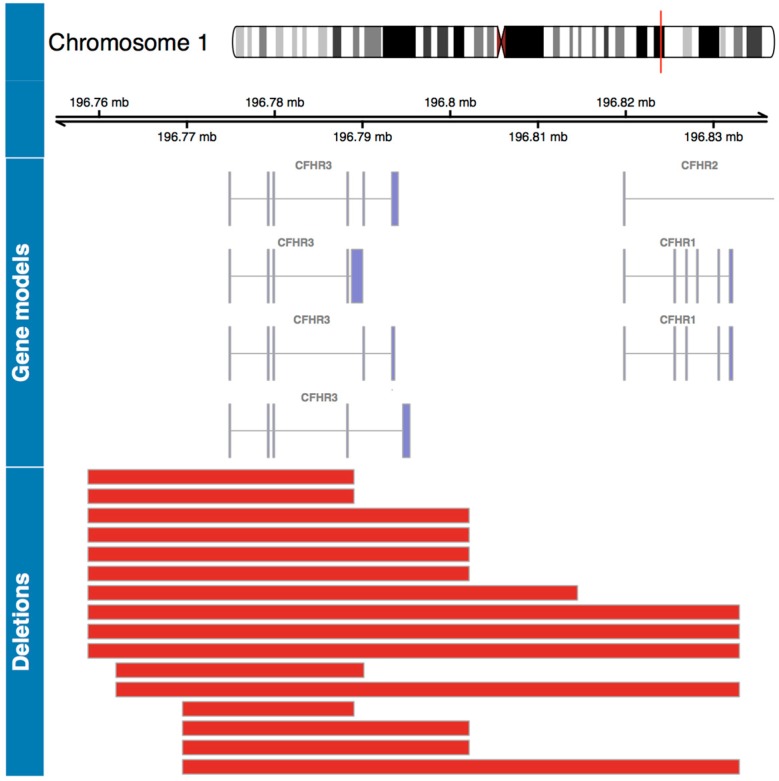
Genomic map for statistically significant deletions in severe patients. The top panel shows the chromosome containing the associated regions with the coordinates, and the subregion of interest marked in red. The second panel shows the gene models for the alternative transcripts of the *CFHR* genes located in the region of interest. The bottom panel shows the size of each CNV deletion along the same genomic coordinate, each red line corresponds to a deletion in a patient.

**Figure 3:**
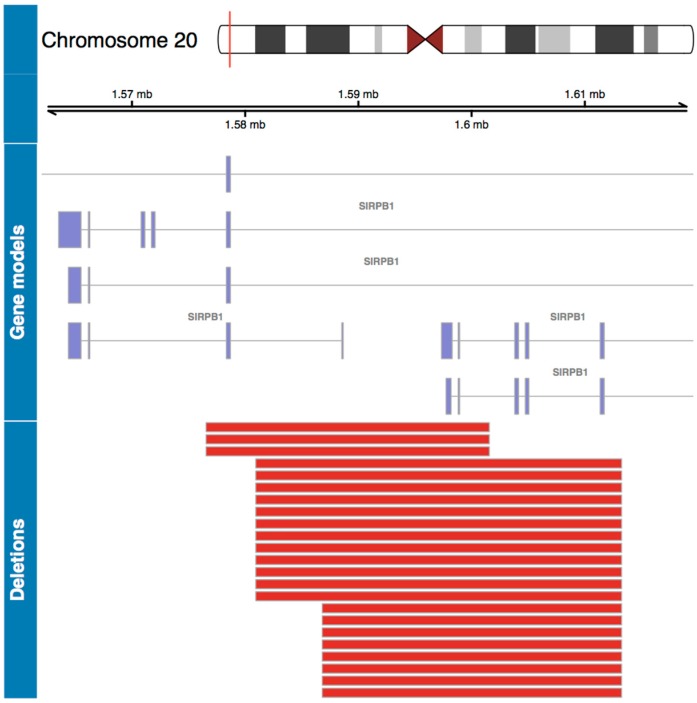
Genomic map for statistically significant deletions in mild patients. Top panel shows the chromosome containing the associated regions with the coordinates, and the region of interest marked in red. The middle panel shows the gene models of the alternative transcripts of the *SIRPB1* gene located in the region of interest. The bottom panel shows the size of each CNV deletion along the same genomic coordinate, each red line corresponds to a deletion in a patient.

A genome wide association analysis was performed, comparing CNV frequencies between severe and mild patients. Three CNVs were significantly enriched in severe patients (CNVs 1 to 3), and three CNVs were significantly enriched in mild patients (CNVs 4 to 6) (Table 2). Of these six CNVs, only four are located in coding regions with a distance to the closest gene = 0 (CNVs 2, 3, 4 and 5), so they can have a potentially disruptive effect on the gene structure and function of the genes that are located in the region. CNVs 3 and 5 were discarded because are duplications that include olfactory receptors, which are known to have a great variation mainly due to their function in the perception of smell [34]. In severe patients, CNV2, which corresponds to a deletion of 19.53 kb located in the q31.3 region of chr1:196769493-196789029, was significantly more frequent in patients with a severe infection (*p* < 0.05, OR = 3.03 IC95 1.04–12.5). Of the 16 severe cases with the deletion, 13 were heterozygous and three homozygous. Figure 2 shows the size of this deletion in different severe patients. This deletion includes two genes of the complement factor H related proteins: *CFHR1* and *CFHR3,* which regulate the complement cascade [35]. As seen in Figure 2, these two genes have different splicing variants (gene models). In different patients this deletion has different sizes, so the product of having the deletion might be different. However, it always includes coding regions (exons) of *CFHR3,* and in five patients it also includes *CFHR1*. CNV4, which corresponds to a deletion of 1.81 kb located in the p13 region of chr20:1599742-1601561 containing signal regulatory protein beta 1 (*SIRPB1)* gene, was significantly more frequent in patients with a mild clinical course (*p* < 0.05, OR = 0.3 IC95 0.12–0.76). The 23 mild cases have a heterozygous deletion. The sizes of these deletions are shown in Figure 3. Since *SIRPB1* has five splicing variants and the deletions have different sizes in different patients, the products of the deletion might be different. However, it always involves at least one exon of the gene, which will produce a truncated protein with reduced function or without function.

## 4. Discussion

In this study, unbiased comparison between the genome variants in ANDV infected patients that had different clinical courses was performed. CNVs were compared, since they are an important source of human variation, and CNVs have been associated with susceptibility to viral infectious diseases such as human immunodeficiency virus (HIV), hepatitis C (HCV) and hepatitis B (HBV). For HIV, a high copy number (>2) of the chemokine *CCL3L1* gene was associated with an 80% reduced risk of acquiring HIV in intravenous drug users [36]. Having less than two copies of this same CNV was found more frequently in HCV infected patients compared to non-infected individuals [37]. In HBV it was found that a CNV containing the *TLR7* gene was associated with chronic HBV infection [38].

**Figure 4:**
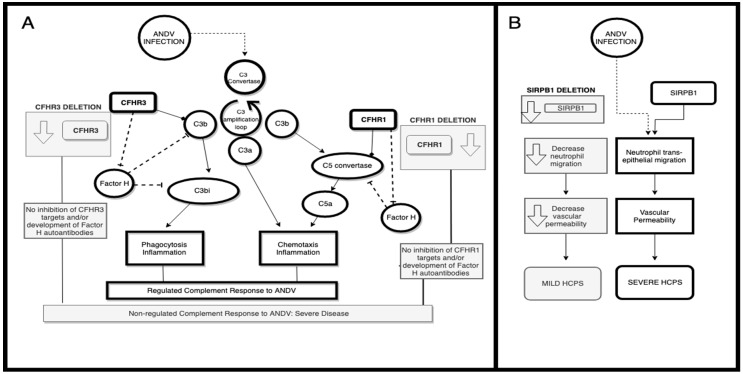
(**A**) model of CFHR1-3 participation in Andes orthohantavirus (ANDV) infection response. In white is the patient’s complement pathway response to ANDV infection. In grey is the proposed effect caused by the *CFHR* genes’ deletion. (**B**) model of *SIRPB1* in ANDV infection response. In white is the neutrophil response to ANDV infection. In grey is the proposed effect caused by *SIRPB1* deletion.

In the current study, when comparing the frequencies of CNVs in mild and severe HCPS patients, we find two CNVs associated with the clinical course of HCPS that contain genes that could be good candidates for participation in the patient’s response to ANDV infection. The first is CNV2, which contains *CFHR3* and *CFHR1* genes. CNV2 showed an OR 3.31 (IC95 1.04–12.55), so patients with this deletion have 3.3 times more risk of developing a severe disease when infected with ANDV. CFHRs can regulate the complement cascade, but there is opposite evidence on how this mechanism works. The complement system is important for host innate and adaptive immunity and mounts a protective immune response to invading microbes [19]. After microbe infection, complement can be activated by three different pathways, and all three converge into the generation of C3 convertase (Figure 4A). C3 convertase cleaves the central complement component C3 into the activation product C3b, which forms together with factor B the C3 convertase (C3bBb). These convertases rapidly cleave further C3 molecules, generate C3a and amplify C3b deposition (C3 convertase amplification loop). C3bi, a subproduct of C3b, is an inductor of phagocytosis and inflammation. Further complement activation leads to C5 convertase formation and cleavage of C5 to C5b and C5a [39,40]. The cleavage products C3a and C5a are potent anaphylatoxins that are chemotactic for immune cells, such as macrophages and neutrophils, and also induce inflammation [39,40,41]. This is a highly regulated pathway, which can be inhibited by factors such as complement factor H. Once activated, this powerful defense system is tightly controlled on host cell surfaces by both membrane-anchored and surface-attached soluble regulators.

CFHRs, which can act as inhibitors of the complement cascade by inhibiting C5 convertase activity and the terminal complex formation [42], when deleted can increase complement signaling. Others have found that CFHRs are enhancers of complement activation by competing with the inhibitory complement factor H and enhance the formation of C3 convertase [43]. In the current study, it was found that total or partial deletion in *CHFR3* and *CHFR1* genes is more frequent in patients with severe disease. This same deletion has been found to be associated with increased risk for a severe renal disease named atypical hemolytic uremic syndrome (aHUS) [44]. Between 11–16% of the patients with aHUS have deletions in *CFHR1* and/or *CFHR3* [45], and approximately two thirds of the patients with the deletion have auto-antibodies to complement factor H [45]. So, in an indirect way, the deletion in the *CFHR1-3* locus can lead to activation of the complement pathway by inhibiting factor H with autoantibodies. It has been shown for hantavirus infection that the complement pathway activation is increased in more severe patients. This is true for Puumala hantavirus (PUUV) infected patients, where an increase in complement pathway activation was found in patients with a severe clinical course [42,46,47]. Moreover, a study performed on ANDV infected patients also showed that complement activation, measured as a decrease in C5/C5a, increases the odds of having a severe disease [15]. In this regard, the deletion we found in severe patients, may be participating in the increase of complement pathway activation observed in patients with this clinical course (Figure 2).

A second finding of this study is the increase in frequency of the deletion CNV4 including the *SIRPB1* gene in mild HCPS patients. CNV4 showed an OR 0.3 (IC95 0.12–0.76), so patients with this deletion have 0.3 times less risk of developing a severe disease when infected with hantavirus. *SIRPB1* codifies a transmembrane protein that mediates the interactions with proteins containing immunoreceptor tyrosine-based activation motifs (ITAM) and is a member of the immunoglobulins superfamily [48,49]. It participates in the innate immune response and has been shown to induce neutrophil transepithelial migration (Figure 4B) [48]. In patients infected with the old world hantavirus, PUUV, neutrophil activation products are elevated in acute disease and positively correlate with kidney dysfunction [50]. In fact, in an severe combined immunodeficient (SCID) mouse model infected with Hantaan hantavirus (HTNV), there was an increase in neutrophils in lungs and blood suggesting that neutrophil response is important in hantavirus infection [51]. Moreover, when neutrophils were depleted, the occurrence of pulmonary edema was inhibited and vascular permeability was also significantly suppressed, showing the importance of neutrophil migration to the site of infection to induce vascular permeability [51]. Since *SIRPB1* promotes neutrophil migration to the site of infection, the CNV deletion containing this gene is a protective factor. The deletion is found more often in patients with mild disease, in whom vascular permeability is less marked than in patients with disease. The absence of *SIRPB1* would prevent neutrophils from invading lung tissue (Figure 4B) and in this way help reduce capillary leakage.

The immune response to hantaviruses is a very complex and highly regulated process. Our results show that there is variation in genes involved in innate immune responses that is associated with disease severity. Although these results do not show mechanisms, they may encourage attempts to confirm them in clinical studies or animal models.

## Figures and Tables

**Table 1:** Hantavirus cardiopulmonary syndrome (HCPS) patients’ demographic characteristics.

		Severe (*n* = 107)	Mild (*n* = 88)	*p*-Value
**Age (years), Mean**		38.12	35.33	0.99 ^a^
**Gender, *n* (%)**	Man	71 (66.4)	62 (70.5)	0.64 ^b^
	Woman	36 (33.6)	26 (29.5)	
	Total	107 (100)	88 (100)	

^a^*t*-test; ^b^ Fisher’s exact test.

**Table 2:** CNV frequencies in HCPS mild and severe patients.

CNV Number	Genome Coordinates (GRCh38/hg38)	OR (95% CI)	*p*-Value	Gain/Loss	Severe (*n* = 60)	Mild (*n* = 51)	* Distance to Closest Gene (bp)	Putative Affected Gene	Cytoband
**CNVs enriched in severe patients**									
CNV1	chr11: 18940271–18940271	4.37 (1.10–25.44)	0.028	Loss	13	3	5269	*MRGPRX1*	11p15.1
CNV2	chr1: 196769493–196789029	3.31 (1.04–12.55)	0.029	Loss	16	5	0	*CFHR1, CFHR2, CFHR3*	1q31.3
CNV3	chr11: 4950775–4955384	3.31 (1.04–12.55)	0.029	Gain	16	5	0	*OR51A2*	11p15.4
**CNVs enriched in mild patients**									
CNV4	chr20: 1599742–1601561	0.3 (0.12–0.76)	0.007	Loss	12	23	0	*SIRPB1*	20p13
CNV5	chr14: 19742838–19939483	0.19 (0.03–0.8)	0.010	Gain	3	11	0	*OR4K1, OR4K2, OR4K5, OR4M1, OR4N2, OR4Q3*	14q11.2
CNV6	chr2: 146106836–146109366	0.16 (0.01–0.85)	0.022	Gain	2	9	478,078	*LOC728773*	2q22.3

* The distance from the CNV to the closest proximal gene annotated. If the value is 0, the CNV resides directly on the gene.

## Material Accesion Number

The dataset is available to download here: https://www.ebi.ac.uk/ena/data/view/PRJEB33276, the study information is available through here: https://www.ebi.ac.uk/eva/?eva-study=PRJEB33276, European Variation Archive EMBL-EBI accession number PRJEB33276.
